# Reduction in Volume of Nucleus Basalis of Meynert Is Specific to Parkinson’s Disease and Progressive Supranuclear Palsy but Not to Multiple System Atrophy

**DOI:** 10.3389/fnagi.2022.851788

**Published:** 2022-04-01

**Authors:** Sophia Rogozinski, Martin Klietz, Gesine Respondek, Wolfgang H. Oertel, Michel J. Grothe, Joana B. Pereira, Günter U. Höglinger

**Affiliations:** ^1^Department of Neurology, Hanover Medical School, Hanover, Germany; ^2^German Center for Neurodegenerative Diseases (DZNE), Munich, Germany; ^3^Department of Neurology, Philipps University of Marburg, Marburg, Germany; ^4^Unidad de Trastornos del Movimiento, Servicio de Neurología y Neurofisiología Clínica, Instituto de Biomedicina de Sevilla, Hospital Universitario Virgen del Rocío/CSIC/Universidad de Sevilla, Seville, Spain; ^5^Division of Clinical Geriatrics, Department of Neurobiology, Care Sciences and Society, Karolinska Institute, Stockholm, Sweden; ^6^Clinical Memory Research Unit, Department of Clinical Sciences, Lund University, Lund, Sweden

**Keywords:** progressive supranuclear palsy, cholinergic innervation, Parkinson’s disease, nucleus basalis of Meynert, voxel-based morphometry, subcortical dementia, multiple system atrophy

## Abstract

**Objectives:**

To study *in vivo* gray matter (GM) volumes of the nucleus basalis of Meynert (nbM) in different parkinsonian syndromes and assess their relationship with clinical variables.

**Methods:**

T1-weighted magnetic resonance images from patients with progressive supranuclear palsy (PSP, *N* = 43), multiple system atrophy (MSA, *N* = 23), Parkinson’s disease (PD, *N* = 26), and healthy controls (HC, *N* = 29) were included. T1-weighted images were analyzed using a voxel-based morphometry approach implemented in the VBM8 toolbox, and nbM volumes were extracted from the spatially normalized GM images using a cyto-architectonically-defined nbM mask in stereotactic standard space. NbM volumes were compared between groups, while controlling for intracranial volume. Further, within each group correlation analyses between nbM volumes and the Mini Mental Status Examination (MMSE), Hoehn and Yahr stage, PSP Rating Scale, Unified Parkinson’s Disease Rating Scale part III and Frontal Assessment Battery scores were performed.

**Results:**

Significantly lower nbM volumes in patients with PSP and PD compared to HC or patients with MSA were found. No significant correlations between MMSE and nbM volumes were detected in any of the subgroups. No significant correlations were found between clinical scores and nbM volumes in PSP or other groups.

**Conclusion:**

nbM volumes were reduced both in PD and PSP but not in MSA. The lack of significant correlations between nbM and cognitive measures suggests that other factors, such as frontal atrophy, may play a more important role than subcortical cholinergic atrophy in PSP patients. These results may indicate that other drug-targets are needed to improve cognitive function in PSP patients.

## Introduction

The nucleus basalis of Meynert (nbM) is located in the basal forebrain and consists of acetylcholine (ACh)–releasing neurons, which provide major cholinergic projections to cortical brain areas and the amygdala (Liu et al., 2015). The nbM plays an important role in memory, attention, perception, and arousal and has been shown to degenerate in multiple neuropsychiatric diseases (Bartus et al., 1982). In fact, Lewy bodies, the most characteristic histopathological feature of Parkinson’s disease (PD) and dementia with Lewy bodies (DLB), were first described in the nbM (Lewy, 1913). Based on post-mortem brain analyses, marked to moderate atrophy of the nbM was established in the 1980ies in a number of neurodegenerative diseases, including Alzheimer’s disease (AD) (Arendt et al., 1983; Doucette et al., 1986; Allen et al., 1988), PD (Rogers et al., 1985), and DLB ([Bibr B3][Bibr B43]), as well as in a few cases with progressive supranuclear palsy (PSP) (Tagliavini et al., 1984; Rogers et al., 1985; Kasashima and Oda, 2003). Unfortunately, post mortem studies are mostly limited to advanced disease stages and do not allow performing clinico-pathological correlations during earlier disease stages. New methods using magnetic resonance imaging (MRI) now provide the means to evaluate gray matter volume changes of the nbM in living patients and correlate them with concomitant clinical data. For instance, previous volumetric MRI studies showed nbM volume loss was a predictor for the later occurrence of cognitive deficits in PD (Schulz et al., 2018; Pereira et al., 2020) and can be useful to predict cognitive outcome after deep brain stimulation in PD patients (Kubler et al., 2021). In other parkinsonian syndromes, including PSP and multisystem atrophy (MSA), nbM volumes have not been studied in living patients yet, and the relationship between nbM degeneration and clinical variables is still unclear in these diseases. While cognitive and behavioral changes are usually less dominant in patients with MSA (Ou et al., 2017; Santangelo et al., 2018), they are very common in PSP patients and usually occur early in the disease course (Hoglinger et al., 2017) being referred to as “subcortical dementia” (Albert et al., 1974). More specifically, executive functions, behavior, verbal fluency, visuospatial functions, and memory are largely affected in patients with PSP (Bak et al., 2006; Gerstenecker et al., 2013; Rittman et al., 2013; Burrell et al., 2014), significantly affecting the quality of life of the patients and their caregivers (Pekmezoviæ et al., 2015). Thus, treatment approaches for cognitive and behavioral deficits are highly needed for patients with PSP, and the nbM with its cholinergic projections could be a potential target.

The aim of the study was to apply MRI volumetric analyses in a large and clinically well characterized cohort of patients with PSP, MSA, PD, and in healthy controls (HC), to assess the amount of nbM atrophy in relation to clinical variables. To our knowledge, this is the first cross-sectional study to describe the relationship between nbM volumes in atypical parkinsonian syndromes and collected clinical parameters in patients with PSP and MSA.

## Materials and Methods

### Participants

Participants were recruited from 2009 to 2013 at the Department of Neurology in Marburg, Germany. Participants with a clinical diagnosis of probable PSP (Litvan et al., 1996), MSA (Gilman et al., 2008), and PD (Gibb, 1988), as well as healthy controls (HC) without neurological or psychiatric disease were included. Ethics approval was obtained at each site from the local ethics committee of the University of Marburg, and all participants gave written informed consent.

### Clinical Assessments

Clinical assessments were performed by experienced movement disorder specialized neurologists from the Department of Neurology at University of Marburg, Germany in all study participants at the time of brain imaging. They included the following scales: Unified Parkinson’s Disease Rating Scale (UPDRS) part III (Goetz et al., 2008), Hoehn and Yahr (H&Y) stage (Martinez-Martin et al., 2018), Mini-Mental State Examination (MMSE) (Folstein et al., 1975) and Frontal Assessment Battery (FAB) (Dubois et al., 2000). In addition, PSP patients also underwent the Progressive Supranuclear Palsy Rating Scale (PSPRS) (Golbe and Ohman-Strickland, 2007), whereas MSA patients also underwent the Unified Multiple System Atrophy Rating Scale (UMSARS) (Wenning et al., 2004). These assessments were performed in the “on” state of the individual patient.

### Magnetic Resonance Imaging Acquisition

MRI was performed in all participants at the time of clinical assessments at University of Marburg, Germany using a 3T Magnetom Trio with a standard CP head coil. T1-weighted 3D magnetization prepared rapid gradient echo (MPRAGE) sequences [Repetition time (TR) 1,900 ms, echo time (TE) 2.52 ms, 1 mm slice thickness, one number of signal averages (NSA) and field of view (FOV) 250 mm^2^, 1 × 1 × 1 mm^3^ resolution], T2- weighted axial and sagittal images, and axial fluid-attenuated inversion recovery images were included in the MRI-protocol.

The MPRAGE sequences were converted to ANALYZE 7.5 format, before pseudonymizing the file names for further processing.

### Magnetic Resonance Imaging Preprocessing

T1-weighted images were preprocessed using the VBM8 toolbox^1^ running under the statistical parametric mapping software (SPM8)^2^ and MATLAB R2016a (The MathWorks, Natick, MA, United States). After reorientation, all images were segmented into gray matter, white matter and cerebrospinal fluid. The resulting gray and white matter tissues of each subject were registered to MNI space using DARTEL (Ashburner and Friston, 2011). Finally, the gray matter tissues were warped using the individual flow fields resulting from the DARTEL registration, and voxel values were modulated for volumetric changes introduced by the normalization. The volumes of two basal forebrain subregions corresponding to Ch1/Ch2 (medial septum/diagonal band of Broca) and Ch4 (nbM) were calculated from the normalized images using the masks of a stereotactic map obtained by combining post-mortem MRI with subsequent histological analysis (Kilimann et al., 2014). In addition, the total intracranial volume (ICV) of each subject was calculated as the sum of gray matter, white matter and cerebrospinal fluid segmentations in order to account for differences in head size in the statistical analyses.

### Statistical Analysis

All statistical calculations were performed with the SPSS 26.0 program (IBM, Armonk, United States). Descriptive data were reported as means, standard deviations and ranges. Analyses of variance and Bonferroni *post-hoc* test were used to compare nbM volumes between groups, while adjusting for ICV. Kruskal-Wallis *H*-test was used to compare FAB, MMSE, disease duration and H and Y between groups. The significance level was set at α < 0.05. Pearson correlations were used to examine the association between nbM volumes and age, PSPRS and UPDRS III, whereas non-parametric Spearman’s rho correlations were used for the following non-normally distributed variables: FAB, MMSE, disease duration, and H&Y. To compensate for multiple comparison (*m* = 16) in these results Bonferroni correction was applied (significance level α = 0.05/16 = 0.003).

## Results

### Study Participants

In total, clinical and imaging data of 121 participants were included in the study. Of these, 26 (21.5%) had a clinical diagnosis of probable PD (Gibb, 1988), 43 (35.5%) a clinical diagnosis of probable PSP (Litvan et al., 1996), 23 (19%) a clinical diagnosis of MSA (Gilman et al., 2008), and 29 (24%) were best possible age-matched individuals, without known neurologic or psychiatric diseases as healthy controls (HC). Demographic and clinical data of all participants are shown in Table 1. Mean age at examination was 63.9 ± 7.5 years, with PSP patients having a significantly higher mean age at examination compared to MSA patients and HC (*P* < 0.05). Mean age at disease onset among all patients was 61.2 ± 6.9 years, with PSP patients having a significantly higher mean age at onset compared to MSA patients (*P* < 0.001). Mean disease duration at the time of examination was 3.7 ± 2.4 years.

**TABLE 1:** Demographic and clinical data of the sample.

	Total	PD	MSA	PSP	HC
N	121	26	23	43	29
M:F [N, (%)]	68:53 [56:44]	20:6 [77:23]	14:9 [61:39]	22:21 [51:49]	12:17 [41:59]
Age at onset [years, mean ± SEM (range)]	61.2 ± 6.9 [42–73]	60.5 ± 8.6 [42–73]	56.8 ± 6.4 [46–72]	64.1 ± 4.3 [50–72]	−
Age at exam [years, mean ± SEM (range)]	63.9 ± 7.5 [41–76]	64.3 ± 9.2 [41–76]	60.4 ± 6.9 [47–75]	67.4 ± 4.6 [53–74]	61.1 ± 7.5 [49–76]
Disease duration (years., mean ± SEM (range)]	3.7 ± 2.4 [0.5–12]	5.1 ± 3.6 [0.5–12]	3.6 ± 1.7 [0.5–7]	3.1 ± 1.5 [1–7]	−
Hoehn&Yahr [mean ± SEM (range)]	3 ± 0.8 [2–4]	2.3 ± 0.5 [2–3]	3 ± 0.6 [2–4]	3.3 ± 0.7 [2–4]	−
MMSE [mean ± SEM (range)]	27.5 ± 2.6 [16–30]	28.3 ± 1.7 [25–30]	27.8 ± 3 [16–30]	26.7 ± 2.7 [17–30]	−
UPDRS III [mean ± SEM (range)]	22.4 ± 5.5 [12–34]	20 ± 5.5 [12–29]	23.6 ± 5 [15–33]	23.2 ± 5.5 [12–34]	−
FAB [mean ± SEM (range)]	14.6 ± 3.5 [6–18]	15.9 ± 2.2 [10–18]	15.5 ± 1.8 [12–18]	11.9 ± 3.3 [6–18]	18 ± 0 (*N* = 16)

*The data are reported as mean, standard deviation and range for healthy controls (HC), Parkinson’s Disease (PD), Multiple system atrophy (MSA), and Progressive supranuclear palsy (PSP). MMSE, Mini mental status examination; UPDRS III, Unified Parkinson’s Disease Rating Scale part III; FAB, Frontal assessment battery.*

### Clinical Scales

Clinical data of all participants are summarized in Table 1. The mean FAB score among all participants was 14.6 ± 3.5. In the PSP group, the FAB score was significantly lower compared to the PD, MSA and HC groups (Kruskal-Wallis test, *p* < 0.0001, *post hoc* Bonferroni PSP vs. PD *p* = 0.0003, PSP vs. MSA *p* = 0.048, PSP vs. HC *P* < 0.0001). In the PD and the MSA groups, the FAB scores were significantly lower compared to the healthy controls (Kruskal-Wallis-Test, *p* < 0.0001, *post hoc* Bonferroni PD vs. HC *p* = 0.027, MSA vs. HC *p* = 0.027).

The median H&Y stage among all participants was 3. The median H&Y stage was significantly lower in the PD group compared to the PSP and MSA groups (Kruskal-Wallis test, *p* < 0.0001, *post hoc* Bonferroni PD vs. MSA *p* = 0.006, PD vs. PSP *p* < 0001).

The mean MMSE score was 27.5 ± 2.6. In the PSP group, the MMSE score was significantly lower compared to the PD group (Kruskal-Wallis test, *p* = 0.01, *post hoc* Bonferroni *p* = 0.033). The mean score in the UPDRS part III among all patients was 27.5 (± 2.6). There were no significant differences in the subgroup analyses (Kruskal-Wallis test, *p* = 0.074). In the PSP group the mean PSPRS score was 42.3 (±8), in the MSA group the mean UMSARS score was 22.3 (±5.6).

### Nucleus Basalis of Meynert Volumes

**FIGURE 1:**
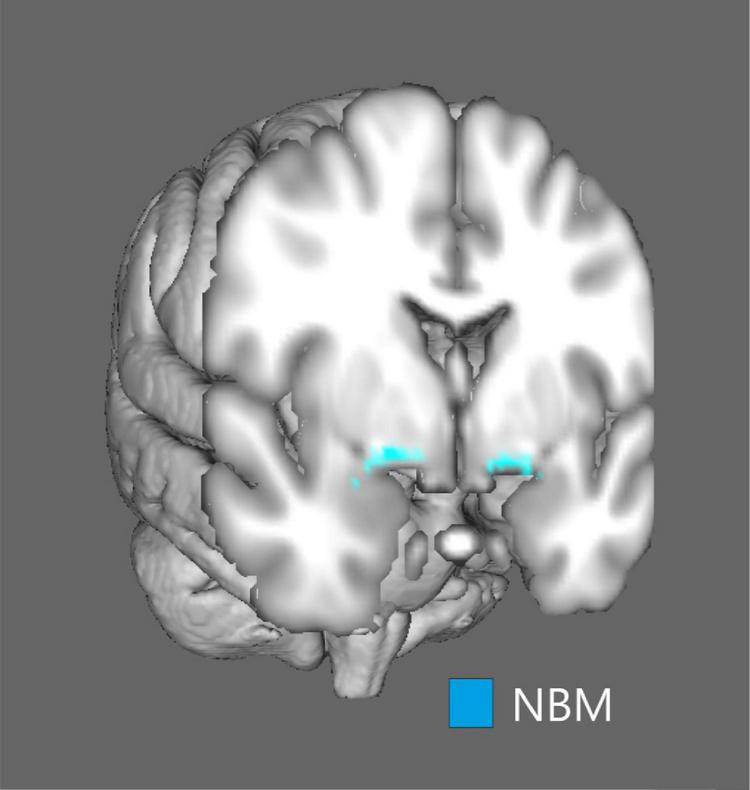
Visual representation of the nucleus basalis of Meynert. The mask of the nucleus basalis of Meynert was overlaid on a structural MRI surface template provided by the fMRI Software Library (FSL) software.

**FIGURE 2:**
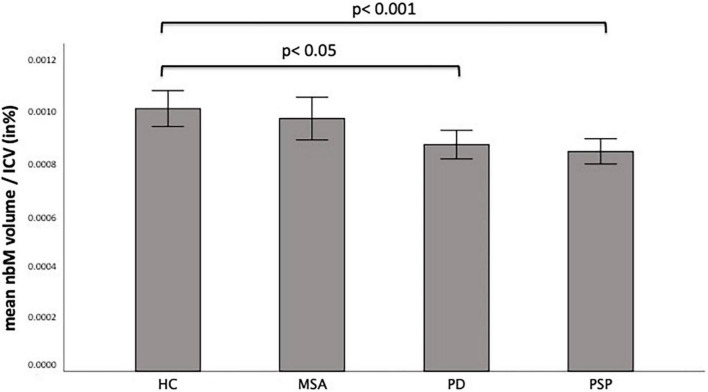
Mean nucleus of basalis Meynert (nbM) volumes corrected for intracranial volume (ICV) per diagnosis. The bar chart displays the nbM volume corrected for the individual ICV in percent in healthy controls (HC) and patients with Parkinson’s disease (PD), Multiple system atrophy (MSA), and Progressive supranuclear palsy (PSP). Significant differences are marked with the corresponding *p*-values.

Figure 1 shows the anatomical localization of the nbM on a structural MRI template. The nbM volumes significantly differed between groups (ANOVA, *p* < 0.001) while controlling for intracranial volume (Figure 2). In PSP patients, nbM volumes were significantly lower compared to HC (Bonferroni *post-hoc* test, *p* < 0.001). Likewise, in PD patients, nbM volumes were significantly lower compared to HC (Bonferroni *post-hoc* test, *p* = 0.023). There was no significant difference in the nbM volumes between PSP and PD patients (Bonferroni *post-hoc* test, *p* = 1.000).

### Volumes of Other Brain Regions

**FIGURE 3:**
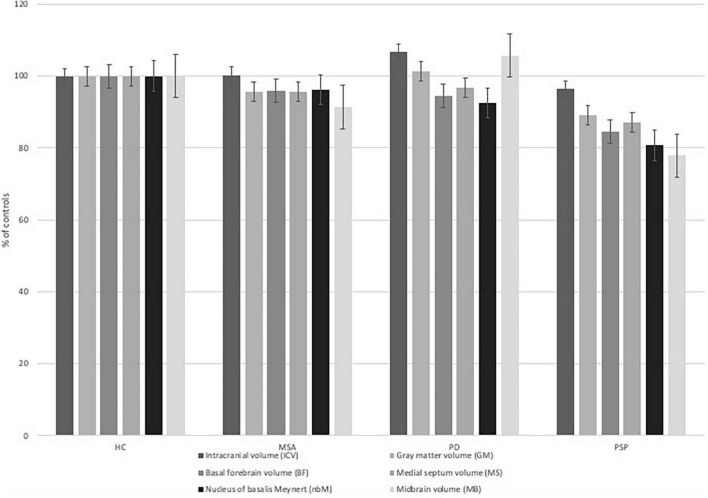
Brain volumes of interest per diagnosis in relation to healthy controls (HC). The bar chart displays different volumes of interest [Intracranial volume (ICV), gray matter volume (GM), basal forebrain volume (BF), medial septum volume (MS), nucleus of Meynert volume (nbM), midbrain volume (MB)] in relations to the volumes measured in healthy controls in percent.

The whole-brain gray matter (GM) volume significantly differed between all subgroups (ANOVA, *p* = 0.001) while controlling for intracranial volume (ICV) (Figure 3). Gray matter volume loss was largest in PSP patients compared to HC (Bonferroni *post-hoc* test, *p* = 0.0004). There was a significant difference in midbrain (MB) volume between all subgroups (ANOVA, *p* < 0.0001) while controlling for ICV.

The MB volume was significantly lower in MSA patients compared to HC (Bonferroni *post-hoc* test, *p* = 0.0005) and PD patients (Bonferroni *post-hoc* test, *p* = 0.003). The MB volume was significantly lower in PSP patients compared HC (Bonferroni *post-hoc* test, *p* < 0.0001), MSA patients (Bonferroni *post-hoc* test, *p* = 0.008), and PD patients (Bonferroni *post-hoc* test, *p* < 0.0001).

### Correlations of Nucleus Basalis of Meynert Volume With Clinical Parameters

All correlations of nbM volume and clinical features are displayed in Table 2. Each individual hypothesis was tested with Bonferroni correction at α < 0.003. There was a trend of a negative correlation between age at examination and nbM volume in the PD group (Pearson correlation coefficient = −0.540, *p* = 0.004), although it did not reach statistical significance after Bonferroni correction. Also, there was no significant correlation between nbM volume and age at examination in the other subgroups (HC: Pearson correlation coefficient = −0.220, *p* = 0.252, MSA: Pearson correlation coefficient = −0.294, *p* = 0.174, PSP: Pearson correlation coefficient = −0.261, *p* = 0.92). There was no significant correlation between disease duration and nbM volume in PSP patients (Spearman’s rho = 0.285, *p* = 0.071).

**TABLE 2:** Correlation of nbM volume and clinical features [healthy controls (HC), Parkinson’s Disease (PD), Multi system atrophy (MSA), Progressive supranuclear palsy (PSP), Mini mental status examination (MMSE), Unified Parkinson’s Disease Rating Scale part III (UPDRS III), Frontal assessment battery (FAB)].

	Age at exam*^c^*	Disease duration*^n^*	UPDRS III*^c^*	MMSE*^n^*	FAB*^n^*
HC	*r* = −0.22	n.a.	n.a.	n.a.	n.a.
PD	*r* = −0.54*	*r* = −0.26	*r* = −0.15	*r* = 0.45	*r* = 0.32
MSA	*r* = −0.29	*r* = 0.15	*r* = 0.01	*r* = 0.40	*r* = 0.06
PSP	*r* = −0.26	*r* = −0.29	*r* = −0.26	*r* = 0.31	*r* = −0.18

*p < 0.05.

*^c^Parametric correlation with Pearson.*

*^n^Non-parametric correlation with spearman-rho.*

There was no significant correlation between nbM volumes and FAB scores in any patient group (MSA: Spearman’s rho = 0.057, *p* = 0.861, PD: Spearman’s rho = 0.318, *p* = 0.139, PSP: Spearman’s rho = −0.180, *p* = 0.292).

There was no significant correlation between nbM volumes and MMSE in any patient group (MSA: Spearman’s rho = 0.396, *p* = 0.068 PD: Spearman’s rho = 0.448, *p* = 0.032 PSP: Spearman’s rho = 0.314, *p* = 0.111).

There was no significant correlation between nbM volumes and any of the motor scales, including H&Y stage (MSA: Spearman’s rho = −0.118, *p* = 0.59, PD: Spearman’s rho = −0.96, *p* = 0.663, PSP: Spearman’s rho = −0.66, *p* = 0.676), UPDRS III scores (MSA: Pearson correlation coefficient = −0.08, *p* = 0.972, PD: Pearson correlation coefficient = −0.149, *p* = 0.497, PSP: Pearson correlation coefficient = −0.257, *p* = 0.131), and PSPRS scores (Pearson correlation coefficient = −0.204, *p* = 0.19) as well as UMSARS scores (Pearson correlation coefficient = −0.208, *p* = 0.293).

## Discussion

In this study, we investigated gray matter volumes of the nbM in patients with PSP, MSA, and PD as well as in HC, using volumetry in T1-weighted MRI, and correlated them with demographic data as well as with motor and cognitive symptoms. While robust MRI data on nbM atrophy in patients with PD were previously published (Ray et al., 2018; Gang et al., 2020), to our knowledge no studies had investigated nbM gray matter volumes in patients with PSP or MSA using MRI. Gray matter volume of the nbM was significantly lower in PSP and PD patients compared to controls. However, in neither subgroup a significant correlation with cognitive or other clinical scores could be detected.

In PD, MSA and PSP patients no significant correlation of disease duration an nbM volume was detected. These findings are in contrast to previously published neuropathological studies where a negative correlation of neuronal loss in the nbM and age at death was found (Tagliavini et al., 1984; Kasashima and Oda, 2003) suggesting that nbM atrophy may not reach significant dimensions in late onset patients. Complementary studies are necessary to confirm this hypothesis. There was no correlation between age and nbM volume in any patient subgroup or HC. But there was a strong trend for a negative correlation in the PD group without a negative correlation with disease duration. Further prospective studies with a larger number of patients are necessary to investigate other factors that may influence nBM atrophy in PD patients, such as age.

In previous studies from our group, gray matter atrophy in PSP was demonstrated predominantly in the midbrain, thalamus and basal ganglia, cerebellum and the frontal cortex. These results were consistent with the literature (Boxer et al., 2017), while in PD there was no pronounced atrophy (Fu et al., 2020). By means of histopathological examinations, a few studies previously demonstrated atrophy of the nbM in small case numbers of deceased PSP patients (Tagliavini et al., 1983, 1984; Rogers et al., 1985; Fu et al., 2020). Thanks to new MRI techniques and voxel-based volumetry it is now possible to perform an *in vivo* examination of the nbM. The nbM is subdivided into the anterior, the intermediate and the posterior nbM and previous studies demonstrated differential atrophy patterns of these subregions of the nbM in PD and AD. Some authors demonstrated a caudal-rostral pattern of cell loss in the nbM in AD (Liu et al., 2015), whereas in PD the intermediate part was most affected by cell loss (Liu et al., 2015). Most neuropathological studies have not investigated the relation between pattern of neuropsychological deficits and atrophy of the different parts of the nbM due to the lack of pre-mortem neuropsychological assessments. In our current study there was no correlation between nbM volume and cognition. However, only the MMSE was used as a screening tool for the detection of cognitive deficits and the FAB as a screening tool for executive dysfunctions. Some authors suggest a relation between pathologies in the anterior part of the nbM and executive dysfunction due to limbic and cortical innervations originating from this part (Liu et al., 2015). Since the occurrence of memory deficits is more pronounced in AD patients than in PD patients, it could be concluded that the posterior part of the nbM is more relevant for the occurrence of memory deficits (Liu et al., 2015). In our analysis, we did not differentiate between the different subregions of the nbM.

Overall, the cholinergic system and associated brain regions have only been investigated by a few studies in PSP patients before (Asahina et al., 1998; Warren et al., 2007a,b). Using PET-CT imaging with a ligand to muscarinic ACh receptors in a cohort of 7 PSP patients, 12 PD patients, and 8 HC, (Asahina et al., 1998) showed no evidence of relevant cholinergic cortical denervation in PSP patients. Using *in vitro* receptor autoradiography Warren et al. (2007b) demonstrated a reduction of M2 and M4 receptors in the posterior caudate and putamen, which could indicate a loss of posterior striatal cholinergic interneurons in PSP patients. Chui et al. (1986) investigated the role of non-dopaminergic systems in particular in the mid-cingulate cortex. In contrast to the previously mentioned studies, they did not find altered M2 or M4 receptor density in the caudate nuclei (Chui et al., 1986).

Unfortunately, trials with cholinesterase inhibitors (Litvan et al., 1989; Fabbrini et al., 2001) have not been successful for PSP patients so far. However, the present study underlines that impairment of the nbM occurs in PSP, which could potentially induce a decrease of ACh in the frontal cortex. However, since cognition did not correlate with the extent of atrophy of the nbM it seems reasonable that these impairments were more likely explained by atrophy of the frontal cortex itself. In contrast to these data, cholinesterase inhibitors have robust effects on cognition in PD patients, where nbM pathology is typically not accompanied by marked frontal cortex atrophy (Hanagasi et al., 2017). Further studies are necessary to confirm this hypothesis. Besides treatment with cholinesterase inhibitors, deep brain stimulation of the nbM came into focus as potentially promising treatment option for cognitive symptoms in patients with AD (Luo et al., 2021), LBD (Maltete et al., 2021), and PDD (Gratwicke et al., 2018). In relation to the data of this study, it can be assumed that this will not be a promising approach in PSP.

### Limitations

In this study patients were included based only on clinical diagnosis and not confirmed by neuropathological examination. Magnet resonance microstructural analysis of the nbM may also help to elucidate pathological processes in this nucleus *in vivo* (Klietz et al., 2021). Further, longitudinal studies are needed to investigate nbM atrophy in PSP patients in the course of the disease.

Because only the MMSE and FAB were available in our cohort, we cannot draw conclusions about the spectrum of cognitive deficits. These screening tools cannot replace extensive neuropsychological testing (Tombaugh and McIntyre, 1992) in particular for executive functions, such as CERAD plus or Wisconsin Card Sorting Test (Lange et al., 2018; Lillig et al., 2021). A relevant limitation of this study is the age at examination. This parameter differs within the subgroups and is significantly higher in PSP patients compared to MSA and HC. An age-matched control group would be necessary to further clarify the findings of this study.

## Conclusion

In conclusion, this study demonstrated a significant reduction of nbM volumes in PSP patients compared to a healthy control group. To our knowledge, this is the first *in vivo* MRI study showing this. A lower nbM volume was not significantly correlated with markers of cognitive performance (MMSE, FAB) in PSP patients. This suggests that atrophy of the nbM, and concomitant reductions in cholinergic innervation of forebrain structures, will only have a minor impact on cognition in PSP patients. Future neuropathological studies should address different pathologic processes in the nbM in different Parkinsonian syndromes and investigate regional specific atrophy in sub-segments of the nbM. Moreover, future imaging studies should use higher field strengths and correlate their findings with more extensive neuropsychological testing.

## Data Availability Statement

The raw data supporting the conclusions of this article will be made available by the authors, without undue reservation.

## Ethics Statement

The studies involving human participants were reviewed and approved by the Ethics committee, University of Marburg. The patients/participants provided their written informed consent to participate in this study.

## Author Contributions

GH and JP: conception and design of the study. MK, GR, WO, and GH: acquisition of the data. SR, GR, MK, JP, and GH: analysis and interpretation of the data. SR, GR, and MK: drafting the manuscript. WO, MG, JP, and GH: revision of the manuscript for important intellectual content. All authors approved to the final version of the manuscript before submission.

## Conflict of Interest

The authors declare that the research was conducted in the absence of any commercial or financial relationships that could be construed as a potential conflict of interest.

## Publisher’s Note

All claims expressed in this article are solely those of the authors and do not necessarily represent those of their affiliated organizations, or those of the publisher, the editors and the reviewers. Any product that may be evaluated in this article, or claim that may be made by its manufacturer, is not guaranteed or endorsed by the publisher.

## Footnotes

1
http://www.neuro.uni-jena.de/

2
https://www.fil.ion.ucl.ac.uk/spm/
